# Midgut Transcriptome of the Cockroach *Periplaneta americana* and Its Microbiota: Digestion, Detoxification and Oxidative Stress Response

**DOI:** 10.1371/journal.pone.0155254

**Published:** 2016-05-06

**Authors:** Jianhua Zhang, Yixi Zhang, Jingjing Li, Meiling Liu, Zewen Liu

**Affiliations:** Key Laboratory of Integrated Management of Crop Diseases and Pests (Ministry of Education), College of Plant Protection, Nanjing Agricultural University, Nanjing, China; Institute of Zoology, Chinese Academy of Sciences, CHINA

## Abstract

The cockroach, *Periplaneta americana*, is an obnoxious and notorious pest of the world, with a strong ability to adapt to a variety of complex environments. However, the molecular mechanism of this adaptability is mostly unknown. In this study, the genes and microbiota composition associated with the adaptation mechanism were studied by analyzing the transcriptome and 16S rDNA pyrosequencing of the *P*. *americana* midgut, respectively. Midgut transcriptome analysis identified 82,905 unigenes, among which 64 genes putatively involved in digestion (11 genes), detoxification (37 genes) and oxidative stress response (16 genes) were found. Evaluation of gene expression following treatment with cycloxaprid further revealed that the selected genes (CYP6J1, CYP4C1, CYP6K1, Delta GST, alpha-amylase, beta-glucosidase and aminopeptidase) were upregulated at least 2.0-fold at the transcriptional level, and four genes were upregulated more than 10.0-fold. An interesting finding was that three digestive enzymes positively responded to cycloxaprid application. Tissue expression profiles further showed that most of the selected genes were midgut-biased, with the exception of CYP6K1. The midgut microbiota composition was obtained via 16S rDNA pyrosequencing and was found to be mainly dominated by organisms from the Firmicutes phylum, among which Clostridiales, Lactobacillales and Burkholderiales were the main orders which might assist the host in the food digestion or detoxification of noxious compounds. The preponderant species, *Clostridium cellulovorans*, was previously reported to degrade lignocellulose efficiently in insects. The abundance of genes involved in digestion, detoxification and response to oxidative stress, and the diversity of microbiota in the midgut might provide *P*. *americana* high capacity to adapt to complex environments.

## Introduction

The insect midgut plays critical roles in digestion and nutrient uptake as well as detoxification and oxidative stress responses. These roles are essential for environmental adaptation. In most insects, digestion occurs mainly in the midgut, where a large portion of the insect’s digestive enzymes are produced and secreted, including proteases, lipases, and carbohydrases [1–3]. The insect midgut is also considered to be the centre of detoxification metabolism and stress response, which include three major interrelated pathways: oxidation-reduction, conjugation and hydrolysis [4, 5]. Generally, cytochrome P450 monooxygenases (P450s) are the most important catalysts of oxidation-reduction reactions and able to detoxify many types of xenobiotics [6–13]. Other oxidation/reduction enzymes, such as superoxide dismutases, catalases and peroxidases, can degrade the byproducts of oxidation-reduction reactions [4]. Glutathione S-transferases (GSTs) are particularly important conjugation enzymes, participating in the detoxification of oxidized lipids and exogenous toxins as well as participating in intracellular transport and hormone synthesis [14, 15]. Detoxification is also carried out via hydrolysis and plays an important role in the degradation of insecticides, such as carboxylesterases (CarEs) catalyzing the hydrolysis of pyrethroids and organophosphates [16]. Other proteins, including cadherins, heat shock proteins (Hsps) and ATP-binding cassette transporters (ABC transporters), are also involved in detoxification metabolism or stress response [17, 18].

The most common symbiont in insects is bacteria, which has been reported to mainly exist in insect guts [19–21]. The microbiota of insects have long been known to play significant roles in food digestion and nutrition, host mating preference, protection against pathogens, resistance against parasitoids and detoxification of noxious compounds [22–26]. For example, the cellulase enzyme produced by gut bacteria facilitates lignin degradation, a process vital for hosts to acquire nutrients [27]. Moreover, the gut microbiota of the coffee berry borer, *Hypothenemus hampei*, are able to mediate caffeine detoxification, which is hypothesized to participate in disrupting herbivory inhibition in plants [28]. The bean bug, *Riptortus pedestris*, can acquire *Burkholderia* from the soil and these bacteria confer the ability to degrade fenitrothion [25]. In addition to participating in digestion and detoxification, gut microbiota can produce siderophores to protect the host insect from pathogens such as *Metarhizium anisopliae* [29]. Thus, a comprehensive understanding of the gut microbiota of insects will facilitate studies on host adaptation to complex environments.

Cockroaches are one of the oldest known winged insects and maintain close contact with humans. Approximately thirty of the over four thousand species of cockroaches found to date are harmful to humans [30–32]. Generally, cockroaches exist in environments with large amounts of toxic substances, including pollutants, microbial toxins, insecticides and other xenobiotics [33–35]. Thus, the detoxification abilities and oxidative stress response of cockroaches are essential for cockroaches to overcome toxic xenobiotics. In addition, cockroaches show an extremely high digestive capability [3, 36–38]. The most common domestic species of cockroaches and a model organism for entomological research, *Periplaneta americana*, has been well-studied. Previous researches mainly focused on the reproduction, digestive characteristics, effects of adipokinetic hormones, sexually dimorphic glomeruli and related interneurons of *P*. *americana* [3,32,39–41]. However, the transcriptomic information from the midgut of *P*. *americana* is insufficient. In this study, in order to understand the abundance of genes involved in digestion, detoxification and response to oxidative stress, and the diversity of microbiota in the midgut of *P*. *americana*, Illumina sequencing and 16S rDNA pyrosequencing were performed to characterize the midgut transcriptome and microbiota in the midgut. The results may provide clues to understand the mechanism of host adaptation to complex environments in *P*. *americana*.

## Materials and Methods

### Insects and Reagents

A colony of *P*. *americana* was purchased from Feitian Medicinal Animal Co. Ltd. (Danyang, Jiangsu, China). The cockroaches were grown on flours of milled corn and bran cob with an unlimited supply of water, at room temperature 26±1°C, humidity 60–70% and 12 h light/12 h dark photoperiod [41–43]. The insects could not contact pesticides through the provided food, water and rearing box.

Acetone (reagent grade) was purchased from Sigma–Aldrich (St. Louis, MO, USA). Cycloxaprid (97%) was kindly provided by Prof. Li Zhong from the Eastern China University of Science and Technology (Shanghai, China).

### Toxicity Bioassay

The 9th instar nymphs of *P*. *americana* were selected for the toxicity bioassay by topical application method [44–46]. Five dilutions of cycloxaprid were made with acetone. After anesthetization with CO_2_, 10 μL of cycloxaprid solution were applied to the intercoxal space of the ventral mesothorax of *P*. *americana* with a pipette, with acetone alone as the control [46, 47]. Each treatment was replicated three times, with thirty cockroaches in each treatment. Mortality was checked 48 h after treatment.

### RNA Extraction and Transcriptome Sequencing

The 9th instar nymphs of *P*. *americana* were surface-washed with 75% ethanol and rinsed with distilled water. Tissues were dissected on ice with sterile needles and forceps. For transcriptomic sequencing, one sample included the midguts from five nymphs at the 9th instar was collected. Total RNA was extracted with Trizol reagent (Life Technologies, USA) according to the manufacturer’s instructions. DNA contaminants were removed by treating RNA extraction products with RNase-free DNase (Ambion, Austin, TX, USA), and then were purified through phenol-chloroform extraction. The quantity and quality of the RNA were checked by agarose gel electrophoresis (1.5% agarose) and spectrophotometry (Nanodrop Technologies, Wilmington, DE, USA). Extracted RNA was stored at -80°C until use.

Library construction was completed by BGI (Shenzhen, China), and Illumina sequencing was performed using an Illumina HiSeq 2000 sequencer (Illumina Inc., San Diego, CA, USA) [41,48,49].

### DNA Extraction and 16S rDNA Pyrosequencing

For 16S rDNA pyrosequencing of microbiota in midguts of *P*. *americana*, one sample included the midguts from five nymphs at the 9th instar was collected and microbes were obtained according to the method described by Walter *et al* [50]. Total microbial DNA from *P*. *americana* midguts were isolated using a PowerSoil DNA Isolation Kit (MO BIO laboratories, San Diego, USA) according to the manufacturer’s protocol. The quantity and quality of the DNA were checked as mentioned above. Extracted DNA was stored at -80°C until use.

The V1–V3 hypervariable 16S rDNA regions were sequenced using a 454 Life Sciences Genome Sequencer FLX Titanium sequencer (GS-Titanium; 454 Life Sciences, Branford, CT, USA) [51, 52].

### Analysis of Transcriptome Sequencing

After transcriptome sequencing, de novo assembly was carried out with Trinity, a short-read assembly programme, after the remove of low-quality reads [53]. All assembled unigenes were BLASTed against NCBI non-redundant (Nr) protein database, Swiss-Prot, the Kyoto Encyclopaedia of Genes and Genomes (KEGG) database and the Cluster of Orthologous Groups (COG) with a cut-off E-value of 10^−5^. Coding regions and sequence directions were determined by the best aligned results. If the results of different databases conflicted with each other, a priority order of NR, Swiss-Prot, KEGG and COG was followed. The expression abundance of unigenes was calculated using the RPKM method (Reads Per Kilobase per Million mapped reads) [54].

### Analysis of 16S rDNA Pyrosequencing

For analyzing the microbiota, chimera sequences were processed with MOTHUR, and raw reads were preliminarily filtered by QIIME [55,56]. Resulting high-quality sequences were clustered into different operational taxonomic units (OTUs) with a 97% similarity cut-off and aligned to the Greengenes database to determine taxonomic assignments [57]. Sequences were assigned to the following levels: phylum, class, order, family and genus. The relative abundances of each taxon were calculated using R (version 3.1.2) based on the number of sequences belonging to each OTU. Rarefaction curves, sample coverage and richness estimators were calculated using MOTHUR.

### Quantitative Real-Time PCR

For tissue expression profile analysis, foregut, midgut, hindgut, fat body, gastric caecum, Malpighian tubule and salivary gland were collected from the 9th instar nymph, and one kind of tissue from five nymphs at the 9th instar was pooled to one sample. To analyze expression induction, cycloxaprid at LD_50_ dose were applied to the 9th instar nymphs, with acetone treatment as control. After 48 h, midguts were collected from the treated and control nymphs, respectively, and the tissues from five cockroaches were pooled to one sample. Three samples for each tissue or each treatment were prepared for total RNA extraction, as mentioned above. Extracted RNA was stored at -80°C until use.

cDNA was synthesized with Superscript III and random hexamers (Invitrogen, Carlsbad, CA, USA) according to the manufacturer’s instructions. Expression profiling and induction expression analysis of seven selected detoxifying and digestive genes was performed using quantitative real-time PCR (qRT-PCR) with the One Step SYBR PrimeScript RT-PCR Kit (Takara, China). For each qRT-PCR experiment, three independent biological replications, analyzed in three technical replications, were measured. The expression level of each gene was calculated relative to the reference genes β-actin and GAPDH according to the 2^-ΔΔCT^ method and a previously described strategy [41,58,59]. All primers for qRT-PCR were designed with Beacon Designer 7.7 (PREMIER Biosoft International, CA, USA) and are listed in S1 Table.

### Statistical Analysis

Toxicity bioassay data were analyzed using Data Processing System (DPS) software [60]. Statistical analysis of all data was performed using SPSS 20.0 (IBM Corporation, USA). One-way analysis of variance (ANOVA) was used to analyze the expression abundance of selected genes in seven tissues and the effects of cycloxaprid (treated vs. control) on the gene expression levels in the midguts of *P*. *americana*. The least significant difference (LSD) test was further used to compare the means of expression abundance of selected genes in different tissues or between treatments and control at *p*< 0.05 or *p*<0.01. Results were shown as the average ± SEM and were considered to be significant at *p*< 0.05 and very significant at *p*<0.01.

## Results and Discussion

### Transcriptome Sequencing and Unigene Assembly

The raw data and assembled data of transcriptome had been deposited in the NCBI database under the accession number of SRX1659265 and GEIF00000000, respectively. Approximately 88,619,510 raw reads were generated from Illumina sequencing of a cDNA library from *P*. *americana* midguts. After clustering and filtering out low quality sequences, approximately 67,183,862 clean reads were obtained, which were further assembled into 161,821 contigs with a mean length of 261 bp and an N50 length of 327 bp (S2 Table). These contigs were assembled into 82,905 longer sequences (14,814 clusters and 68,091 singletons) with a mean length of 462 bp and the N50 length of 631 bp, which were defined as unigenes [49, 61]. Among these unigenes, 9.21% of the transcriptome assembly was over 1,000 bp (S2 Table).

### Homology Analysis and Gene Ontology (GO) Classification

With a cut-off E-value of 10^−5^, 24,827 from 82,905 unigenes were matched by the Blastx homology search to entries in the NCBI non-redundant (Nr) protein database. The highest match percentage is to *Tribolium castaneum* (11.96%), followed by *Pediculus humanus corporis* (10.11%), *Megachile rotundata* (5.83%), *Acyrhosiphum pisum* (5.24%), *Nasonia vitripennis* (4.99%), *Camponotus floridana*(4.44%), and *Harpegnathos saltator* (3.98%) (S3 Table).

**Fig 1:**
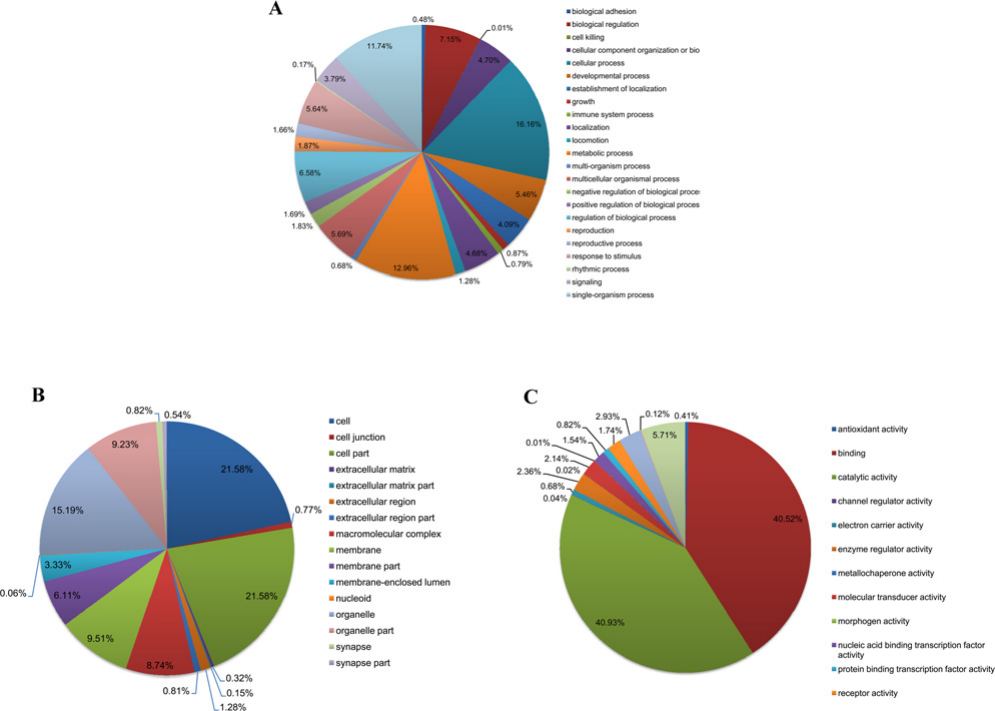
Gene ontology (GO) classification of the *P*. *americana* midgut transcriptome. Unigenes are classified into three main categories: biological process (A), cellular component (B) and molecular function (C).

To further elucidate the functions of these unigenes, Gene Ontology (GO) assignments were used to classify 82,905 unigenes into different functional groups according to GO category [62]. Based on sequence homology, 10,940 unigenes (13.20%) were annotated and classified into one or more functional groups corresponding to the three biological processes (Fig 1). Ultimately, 41,250 annotation hits were aligned to biological process, 23,363 to cellular components, and 13,701 to molecular functions. Among 10,940 annotated unigenes, more than half were aligned to cellular process (60.94%), binding (50.74%), and catalytic activity (51.26%).

### Identification of Putative Genes Related to Detoxification, Digestion and Oxidative Stress Response

**Fig 2:**
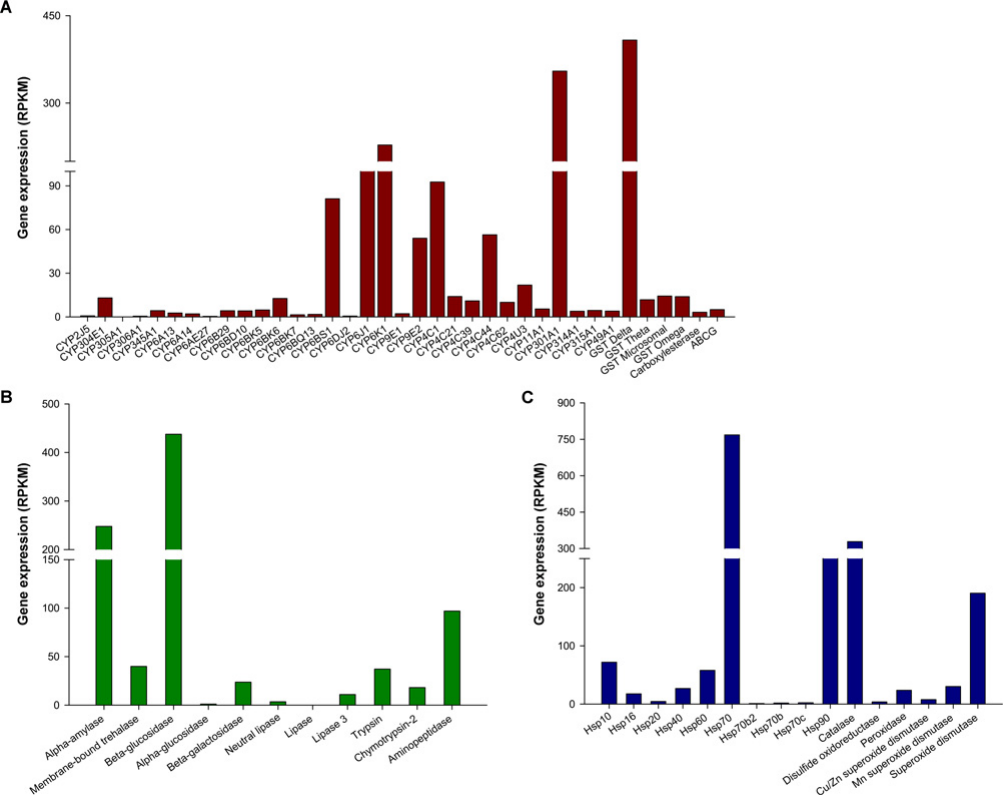
Transcriptomic abundances of sixty-four genes putatively involved in detoxification (A), digestion (B) and oxidative stress response (C). Expression abundance of each genes is indicated by RPKM (Reads Per Kilobase per Million mapped reads) values.

*P*. *americana* maintains close contact with humans and exists in environments with abundant toxic substances [34]. In the current study, the transcriptomic database of the *P*. *americana* midgut is mined to understand the high capability of insects in digestion, detoxification and oxidative stress response. Sixty-four genes were identified to be putatively involved in digestion, detoxification, and oxidative stress response via Blastx homology search with a cut-off E-value of 10^−5^. A total of thirty-seven putative detoxification genes were identified, including thirty-one P450s, four GSTs, one CarE, and one ABC transporter (Table 1). Eleven putative digestive genes were identified, including five carbohydrases, three lipases, and three proteinases (Table 2). Sixteen putative genes related to oxidative stress response were also obtained (Table 3). The abundances of the sixty-four genes in the transcriptome are shown in Fig 2.

**Table 1:** Putative enzymes involved in detoxification that were identified in *P*. *americana* midgut transcriptome.

Name	Gene ID	Length (bp)	Putative identification	Species	Acc. number	Score	E-value
P450s (CYP2)	Unigene42978	227	CYP2J5	*Strongylocentrotus purpuratus*	XP_794251.3	55.8	7.00E-07
	Unigene482	1582	CYP304E1	*Tribolium castaneum*	EEZ99196.1	423.7	1.00E-116
	Unigene20298	1731	CYP305A1	*Tribolium castaneum*	EFA01265.1	454.5	8.00E-126
	Unigene46294	629	CYP306A1	*Manduca sexta*	ABC96068.1	215.7	1.00E-54
P450s (CYP3)	Unigene59287	512	CYP345A1	*Tribolium castaneum*	EFA12856.1	180.6	2.00E-44
	CL2311.Contig1	1576	CYP6A13	*Nasonia vitripennis*	XP_001599214.2	403.7	1.00E-110
	Unigene41639	241	CYP6A14	*Nasonia vitripennis*	XP_001604822.1	58.9	8.00E-08
	CL991.Contig2	237	CYP6AE27	*Zygaena filipendulae*	ACZ97416.2	55.5	9.00E-07
	Unigene53000	218	CYP6B29	*Spodoptera litura*	ADA68173.1	82.8	5.00E-15
	Unigene33798	1505	CYP6BD10	*Laodelphax striatella*	AFU86445.1	416	2.00E-114
	Unigene59250	365	CYP6BK5	*Tribolium castaneum*	EFA12633.1	147.5	1.00E-34
	Unigene14721	410	CYP6BK6	*Tribolium castaneum*	EFA12632.1	152.1	7.00E-36
	CL5642.Contig2	331	CYP6BK7	*Tribolium castaneum*	EFA12631.1	89.7	4.00E-17
	Unigene17337	299	CYP6BQ13	*Tribolium castaneum*	EEZ99338.1	140.6	2.00E-32
	Unigene15697	646	CYP6BS1	*Tribolium castaneum*	EEZ99243.1	166.8	7.00E-40
	Unigene39411	228	CYP6DJ2	*Dendroctonus ponderosae*	AFI45041.1	57.4	2.00E-07
	CL6126.Contig2	1843	CYP6J1	*Blattella germanica*	Q964R1.1	528.9	3.00E-148
	CL104.Contig5	2479	CYP6K1	*Blattella germanica*	Q964R0.1	824.7	0
	CL5911.Contig2	1355	CYP9E1	*Diploptera punctata*	AAR97606.1	565.5	2.00E-159
	Unigene26161	1914	CYP9E2	*Blattella germanica*	Q964T2.1	776.2	0
P450s (CYP4)	Unigene33391	2270	CYP4C1	*Blaberus discoidalis*	P29981.1	891	0
	Unigene49581	1438	CYP4C21	*Blattella germanica*	Q964T1.1	459.1	2.00E-127
	CL6424.Contig2	2032	CYP4C39	*Carcinus maenas*	JC8026	495.7	3.00E-138
	CL3839.Contig1	241	CYP4C44	*Reticulitermes flavipes*	ABB86767.1	138.7	8.00E-32
	Unigene33799	2311	CYP4C62	*Laodelphax striatella*	AFU86425.1	107.1	4.00E-21
	Unigene7659	1637	CYP4U3	*Reticulitermes flavipes*	ABB86762.2	481.5	5.00E-134
P450s (Mitochondrial)	Unigene49004	200	CYP11A1	*Culex quinquefasciatus*	XP_001847403.1	65.1	1.00E-09
	CL5257.Contig1	1891	CYP301A1	*Tribolium castaneum*	EFA02906.1	471.1	9.00E-131
	Unigene53425	509	CYP314A1	*Laodelphax striatella*	AFU86480.1	142.9	5.00E-33
	Unigene56311	1466	CYP315A1	*Apis florea*	XP_003698627.1	453.8	1.00E-125
	Unigene9903	252	CYP49A1	*Apis florea*	XP_003693990.1	63.2	4.00E-09
GSTs	Unigene34767	798	Delta	*Cryptocercus punctulatus*	AFK49803.1	249.6	1.00E-64
	CL6198.Contig1	3264	Theta	*Locusta migratoria*	AEB91980.1	285	1.00E-74
	CL6536.Contig3	4547	Microsomal	*Nilaparvata lugens*	AFJ75808.1	197.6	5.00E-48
	Unigene14342	564	Omega	*Nilaparvata lugens*	AFJ75806.1	120.2	5.00E-26
CarEs	Unigene33193	372	CarE	*Laodelphax striatella*	ADR73024.1	146.4	4.00E-34
ABC transporters	Unigene26028	3120	ABCG	*Nasonia vitripennis*	XP_003426604.1	913.3	0

**Table 2:** Putative enzymes involved in digestion that were identified in *P*. *americana* midgut transcriptome.

Name	Gene ID	Length (bp)	Putative identification	Species	Acc. number	Score	E-value
Carbohydrases	Unigene31916	1952	Alpha-amylase	*Blattella germanica*	ABC68516.1	666.4	0
	Unigene16802	2592	Membrane-bound trehalase	*Bemisia tabaci*	AFV79627.1	881.3	0
	Unigene20412	1707	Beta-glucosidase	*Neotermes koshunensis*	BAB91145.1	658.7	0
	CL1697.Contig2	5600	Alpha-glucosidase	*Harpegnathos saltator*	EFN85516.1	483	8.00E-134
	CL5753.Contig1	2403	Beta-galactosidase	*Camponotus floridanus*	EFN73255.1	693.7	0
Lipases	CL4999.Contig2	711	Neutral lipase	*Danaus plexippus*	EHJ73093.1	211.5	3.00E-53
	Unigene19384	1103	Lipase	*Aedes aegypti*	XP_001654155.1	254.6	6.00E-66
	Unigene19398	1578	Lipase 3	*Acromyrmex echinatior*	EGI70294.1	156	5.00E-36
Proteinases	Unigene13217	950	Trypsin	*Blattella germanica*	AAZ78212.1	237.3	8.00E-61
	Unigene55680	836	Chymotrypsin-2	*Culex quinquefasciatus*	XP_001861618.1	232.3	2.00E-59
	Unigene33558	3041	Aminopeptidase	*Harpegnathos saltator*	EFN87052.1	726.5	0

**Table 3:** Putative enzymes involved in oxidative stress response that were identified in *P*. *americana* midgut transcriptome.

Name	Gene ID	Length (bp)	Putative identification	Species	Acc. number	Score	E-value
Hsps	Unigene20081	751	Hsp10	*Apis florea*	XP_003691248.1	178.3	3.00E-43
	Unigene11971	838	Hsp16	*Pediculus humanus corporis*	XP_002425729.1	141	6.00E-32
	CL509.Contig1	2408	Hsp20	*Locusta migratoria*	ABC84493.1	313.9	2.00E-83
	Unigene5620	1854	Hsp40	*Locusta migratoria*	ABC84495.1	608.6	3.00E-172
	Unigene24161	2524	Hsp60	*Schistocerca gregaria*	AEV89752.1	954.1	0
	CL4484.Contig1	2427	Hsp70	*Cryptocercus punctulatus*	AFK49798.1	1261.5	0
	Unigene12491	519	Hsp70b2	*Tribolium castaneum*	XP_973521.1	302	8.00E-81
	Unigene1076	623	Hsp70b	*Paratlanticus ussuriensis*	AGG36437.1	395.6	8.00E-109
	Unigene19495	1081	Hsp70c	*Paratlanticus ussuriensis*	AFP54305.1	515.4	2.00E-144
	Unigene16700	2780	Hsp90	*Paratlanticus ussuriensis*	AFP54306.1	1361.7	0
Oxidation/reduction enzymes	Unigene22150	3016	Catalase	*Reticulitermes flavipes*	AFV36369.1	987.3	0
	Unigene24256	547	Disulfide oxidoreductase	*Culex quinquefasciatus*	XP_001864945.1	200.7	2.00E-50
	Unigene15114	5083	Peroxidase	*Apis florea*	XP_003694462.1	723	0
	Unigene26872	726	Cu/Zn superoxide dismutase	*Brachymyrmex patagonicus*	ADX36418.1	184.1	5.00E-45
	Unigene31254	1429	Mn superoxide dismutase	*Bombyx mori*	NP_001037299.1	332.4	3.00E-89
	Unigene31277	1024	Superoxide dismutase	*Schistocerca gregaria*	AEV89750.1	267.7	6.00E-70

### Detoxifying Enzymes

Cytochrome P450s (P450s), one of the largest representative families in the *P*. *americana* midgut, play a critical role in insecticide/xenobiotic metabolism and detoxification in all living organisms [6, 63, 64]. P450s are mainly divided into four clades: CYP2, CYP3, CYP4 and mitochondrial CYP [65]. Previous studies have reported that high expression of P450s allows insects to metabolize nearly all classes of insecticides and other xenobiotics, and consequently resulted in high insecticide resistance in many insect species [7–10]. In the transcriptomic database generated in this study, thirty-one P450s were assigned well to appropriate P450 clades according to the Nr annotation, including two in CYP2, sixteen in CYP3, six in CYP4, and five in mitochondrial clade (Table 1). More than half P450s were assigned to CYP3 clade, which agreed with results observed in other insect species [6, 9]. Seven transcripts (CYP301A1, CYP6K1, CYP6J1, CYP4C1, CYP6BS1, CYP4C44, and CYP9E2) were more abundant than the other P450 genes, suggesting that these seven P450 genes might play important roles in insecticide/xenobiotic metabolism or other physiological and biochemical processes in *P*. *americana* midguts (Fig 2A).

GSTs are multifunctional conjugation enzymes and can catalyze the conjugation of reduced glutathione (GSH) with oxidized lipids and exogenous toxins, making the toxins less toxic, more water-soluble and easier to excrete [14]. Several previous studies have shown that increased GST activity resulted in enhanced insecticide resistance in insects [66, 67]. Insect GSTs can be divided into seven classes: Delta, Epsilon, Omega, Sigma, Theta, Zeta and Microsomal, among which the Epsilon and Delta classes were insect-specific and contributed to environmental stress responses, especially during xenobiotic detoxification [68]. In the present study, four GSTs were obtained and assigned to the Delta, Theta, Omega, and Microsomal classes (Table 1). Among four GSTs identified, the Delta GST was the most abundant (Fig 2A), indicating the important role of GSTs from Delta class in xenobiotic metabolism.

CarEs and ABC transporters are also involved in the metabolic activation or detoxification of various drugs, carcinogens and environmental toxicants [69–72]. In this study, one CarE and one ABCG were observed (Table 1). However, the relative abundances of these two genes were low (Fig 2A).

### Digestive Enzymes

The digestive enzymes of insects consist mainly of carbohydrases, lipases and proteinases [3, 73, 74]. The majority of digestive enzymes are produced in the midgut, gastric caeca and salivary glands, and these enzymes can somehow be transported to other tissues such as the foregut [3]. In our transcriptomic database, eleven digestive enzymes were identified, including carbohydrases (alpha-amylase, membrane-bound trehalase, beta-glucosidase, alpha-glucosidase, and beta-galactosidase), lipases (neutral lipase, lipase, and lipase 3), and proteinases (trypsin, chymotrypsin-2, and aminopeptidase) (Table 2). This observation is consistent a previous study regarding digestive enzymes in *P*. *americana* [3]. Three genes (alpha-amylase, beta-glucosidase, and aminopeptidase) were more abundant than other digestive enzyme genes (Fig 2B). These results indicated that active digestive processes were underway in the *P*. *americana* midgut. In addition to digestive functions, aminopeptidases can also detoxify Bt Cry toxins, mycotoxins, organophosphonates, pyrethroid esters, and microbial as well as botanical pesticides [75–77], implying that digestive enzymes might be involved in the positive response towards xenobiotics in insects.

### Enzymes Related to Oxidative Stress Response

Hsps play key roles in various biological and physiological processes, including folding and unfolding of proteins, preventing aggregation of denatured proteins, and detoxifying heavy metals [18, 78, 79]. In the present study, ten Hsps (Hsp10, Hsp16, Hsp20, Hsp40, Hsp60, Hsp70, Hsp70b2, Hsp70b, Hsp70c, and Hsp90) were obtained according to the Nr annotation (Table 3). The majority of these Hsps were Hsp70 genes (4/10) or small Hsp genes (3/10), a profile similar to the common cutworm, *Spodoptera litura* [80]. Hsp70 and Hsp90 were highly abundant in this transcriptome (Fig 2C). The Hsp70 family, the most pervasive Hsps, prevents indiscriminant protein aggregation by tightly binding to denatured proteins under conditions of stress [81]. A previous study of *Drosophila melanogaster* showed that upregulated expression of Hsp70 was closely associated with cold exposure or cold acclimation [82]. Under normal physiological conditions, Hsp90 is an abundant protein that is essential for cold survival during insect diapause [83]. Small Hsps are a family of molecular chaperones that have been extensively studied in insects recently. Small Hsps were upregulated in response to environmental stresses such as thermal stress [84]. In addition to Hsps, six oxidation/reduction enzymes were found in database: catalase, disulphide oxidoreductase, peroxidase, and three superoxide dismutases (Table 3). These oxidation/reduction enzymes could degrade reactive oxygen species, including hydroxyl radicals, hydrogen peroxide, and superoxides [4]. Catalase and superoxide dismutase had relatively high abundances (Fig 2C), suggesting that *P*. *americana* may possess high capacity to overcome complex environmental stresses.

### Expression Regulation of Some Genes by Insecticide Cycloxaprid

The toxicity of cycloxaprid, a novel neonicotinoid insecticide with high insecticidal activities against a range of insect species [85], against *P*. *americana* was tested. The toxicity regression equation was computed as y = 2.3031+1.9996x (r = 0.9906). Based on this toxicity regression equation, the calculated LD_50_ value of the 9th instar nymphs was 22.32 μg/pest (95% CI 19.25–25.89).

**Fig 3:**
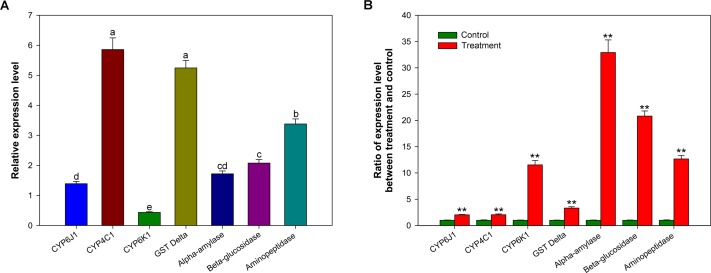
Relative expression levels of seven selected genes (A) and fold changes in the expression of these genes after cycloxaprid treatment (B) in the *P*. *americana* midgut. In (A), different letters indicate significant differences at *p*< 0.05 level among genes. In (B), stars (**) indicate significant differences between the control and cycloxaprid treatment at *p <* 0.01 level.

To evaluate the response to insecticide pressure at the transcriptional level, changes in the expression of seven genes (CYP6J1, CYP4C1, CYP6K1, Delta GST, alpha-amylase, beta-glucosidase and aminopeptidase) in *P*. *americana* midguts after cycloxaprid treatment at the LD_50_ dose was determined. Before the test of expression levels of above genes, an alignment analysis of deduced amino acid sequences was performed through comparing with sequences from other insect species. High similarities among aligned sequences and the conservation in important motifs indicated the annotations to these genes were appropriate (S1, S2, S3, S4 and S5 Figs). The genes were selected based on their expression abundances in the transcriptome. Compared to a control, all selected genes were upregulated to at least 2.0-fold at the transcriptional level. Four genes (CYP6K1, alpha-amylase, beta-glucosidase and aminopeptidase) were upregulated more than 10.0-fold (Fig 3B). These results suggested that these genes might be associated with insecticide (e.g., cycloxaprid) metabolism, which was in agreement with previous reports. For example, studies have found that the CYP3 and CYP4 clades play roles in insecticide/xenobiotic metabolism and that the overexpression of CYP3 or CYP4 genes, such as CYP6ER1, CYP6AY1, CYP6G1, CYP4C27, and CYP4G19, can result in high insecticide resistance to neonicotinoid insecticides [8, 86–89]. A previous study also reported that Delta GST was involved in xenobiotic detoxification [68]. An interesting finding was that the fold increase of three digestive enzyme genes was higher than that of detoxification enzyme genes (Fig 3B). Aminopeptidases have been found to participate in the detoxification of many types of toxins [75–77], indicating that these digestive enzymes might also be involved in the positive response to cycloxaprid application in insects. These results have prompted us to study the roles of digestive enzymes, especially alpha-amylase and beta-glucosidase, in insect responses to the application of insecticides and xenobiotics in future.

### Analysis of Tissue Expression Profiles

To investigate the general expression profiles of seven selected genes, we employed qRT-PCR to determine mRNA levels in various tissues (foregut, midgut, hindgut, fat body, gastric caecum, Malpighian tubule and salivary gland). From the transcriptome database, the selected genes were found to differ in expression abundance. Their abundances were confirmed by qRT-PCR, with exception of CYP6J1 and CYP6K1 (Fig 3A). The result indicates that the transcriptome generally reflects the expression abundances of most genes, albeit incompletely and with some admissible errors. This result was in line with previous reports [47, 61]. The qRT-PCR results showed that CYP4C1, Delta GST and aminopeptidase were more abundant than other selected enzyme genes (Fig 3A), suggesting the pivotal roles that these enzymes play in the *P*. *americana* midgut.

**Fig 4:**
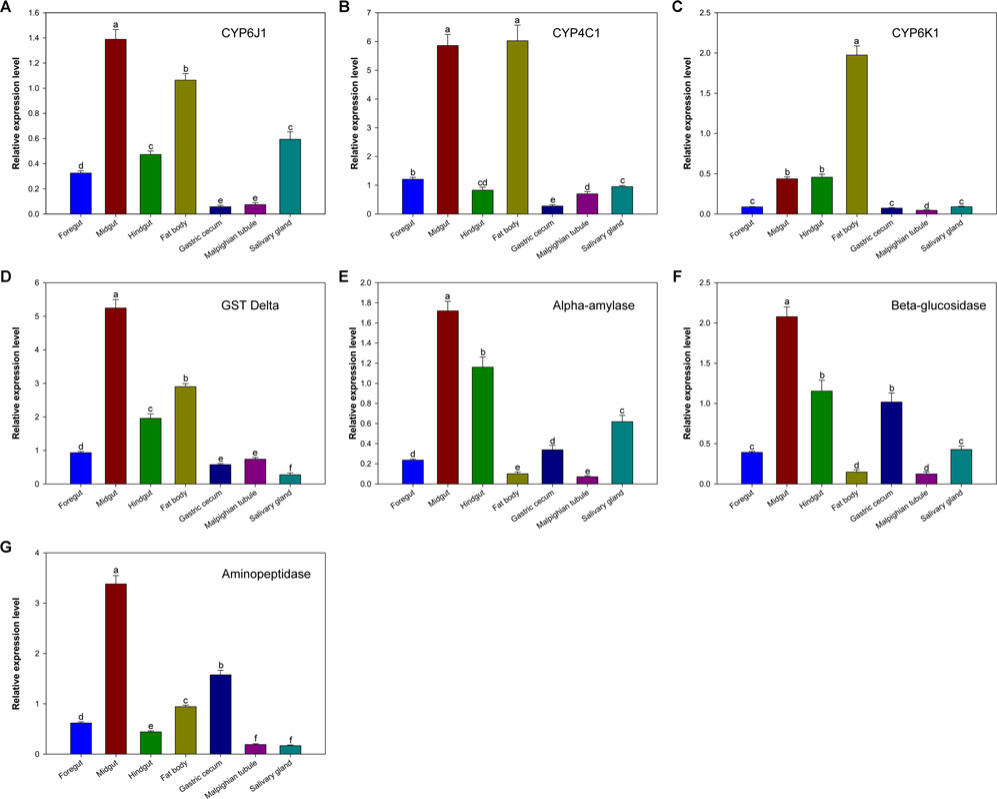
Relative expression levels of seven selected genes in various *P*. *Americana* tissues. Different letters show significant differences among tissues for each gene at *p <* 0.05 level.

Four selected detoxifying enzymes (CYP6J1, CYP4C1, CYP6K1, and Delta GST) were more highly expressed in the midgut and fat body than that in other tissues with the exception of CYP6K1, which was only slightly expressed in the midgut (Fig 4A–4D). This result indicates the important role of the midgut and fat body in detoxification. In insects, detoxification and defence functions mainly proceed in the midgut and fat body and serve to help the insect cope with complex environments. For instance, the major enzyme involved in the primary detoxification pathway of insecticides and other exogenous compounds is mainly found in the midgut and fat body of the cotton bollworm (*Helicoverpa armigera*) [90]. These detoxifying enzymes were also detected in the other five tissues investigated, albeit at much low abundances (Fig 4A–4D). In the hindgut and Malpighian tubule, Delta GST was more highly expressed than the three other detoxifying enzyme genes. Because they are parts of the insect excretory system, the hindgut and Malpighian tubule are mainly involved in maintaining homeostasis and waste elimination [91]. Thus, Delta GST may play a role in the excretion of toxic compounds in the hindgut and Malpighian tubule.

The expression levels of the selected digestive enzymes (alpha-amylase, beta-glucosidase and aminopeptidase) were much higher in the midgut than in other *P*. *americana* tissues (Fig 4E–4G). This result agrees with previous studies, which reported that the main digestive enzymes are produced and secreted in insect midguts [3]. In general, these genes were highly expressed in the hindgut, gastric caecum and salivary gland. Expression of aminopeptidase in the salivary gland was low (Fig 4E–4G). These results imply that the gastric caecum and salivary gland are involved in the secretion of digestive enzymes in *P*. *americana*. In addition, it has been proposed that these digestive enzymes were mainly secreted in the midgut or in the gastric caeca, and transported to the foregut by counter current fluxes and peristaltism and to the hindgut by the normal traffic of food along the gut according to previous studies [3, 92], although their transcriptional expression was also detected in the foregut and hindgut. The abundances of three selected digestive enzymes were lower in Malpighian tubule than that in other tissues (Fig 4E–4G).

### Basic Statistics of Bacterial Communities in *P*. *americana* Midguts

Microbes in insect midguts could aid the insect in responding to pressures from food ingestion, invasion of exogenous microorganisms, insecticide exposure and other external threats [23–26]. Therefore, the bacterial communities in the *P*. *americana* midgut were analyzed via 16S rDNA pyrosequencing.

**Fig 5:**
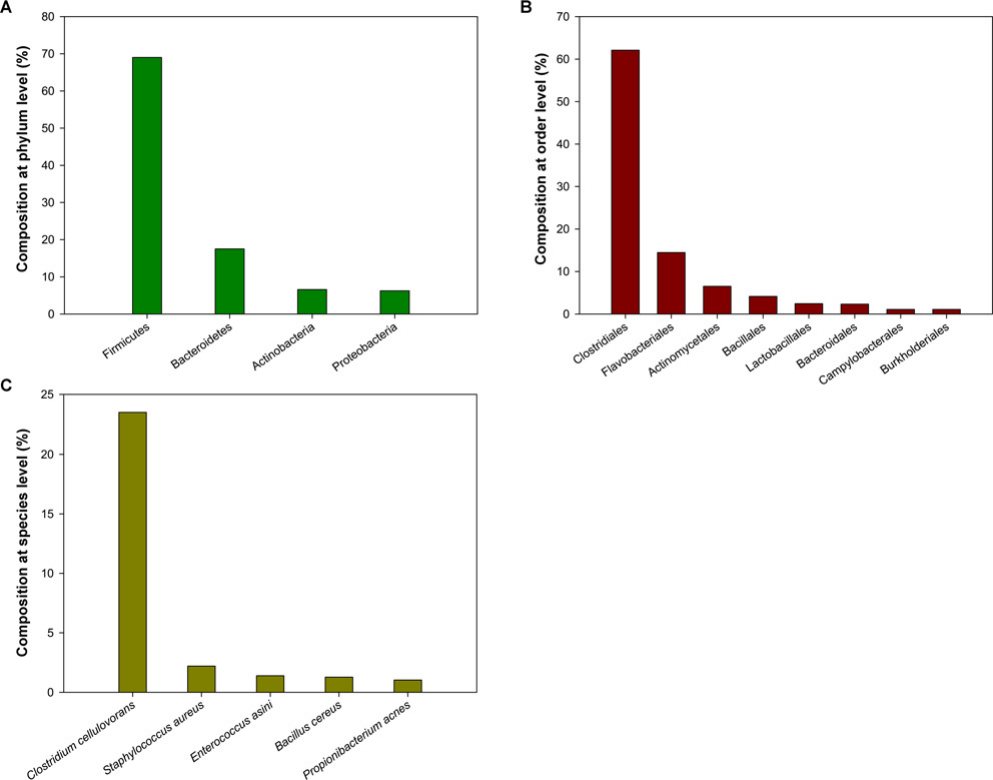
Microbial composition in *P*. *americana* midgut at the phylum level (A), at the order level (B), and at the species level (C). Detected taxa which have more than 1.0% relative abundance in each level are shown.

A total of 27,451 high-quality sequences were obtained after chimaera checking and a strict quality control process. The sequences had an average length 492 bp. The richness estimators and rarefaction curves suggest that the current analysis captured the most dominant phylotypes. Based on a 97% identity, 514 operational taxonomic units (OTUs) were obtained. The species composition of the bacterial community in *P*. *americana* midguts was calculated for various classification levels (phylum, class, order, family, and genera) according to Greengene database. At the phylum level, the composition of the microbiota mainly contained four phyla: Firmicutes, Bacteroidetes, Actinobacteria, and Proteobacteria. The microbiota was dominated by Firmicutes, which represented 69.02% of total sequences (Fig 5A). This result was in agreement with a previous study of the Diamondback moth (*Plutella xylostella*) midgut microbiota, which was also dominated by these four phyla [19]. At the order level, Clostridiales was the most dominant microbiota, comprising approximately 62.10% of total sequences. The Flavobacteriales, Actinomycetales, Bacillales, Lactobacillales, Bacteroidales, Campylobacterales and Burkholderiales orders were also detected (Fig 5B). Some orders have been previously shown to play pivotal roles in detoxification or digestion. For example, in the termite *Reticulitermes flavipes* hindgut, Clostridiales have been shown to play a vital role in the breakdown of lignocelluloses [93]. Detoxifying genes have been acquired by insects via symbionts such as Burkholderiales and *Pseudomonas* over the course of evolution [25, 28]. Lactobacillales was shown to potentially play a role in conferring *P*. *xylostella* resistance to toxins such as the insecticides fipronil and chlorpyrifos [19]. It is noteworthy that *Clostridium cellulovorans* is the most preponderant species in the *P*. *americana* midgut (Fig 5C), which contains a cellulosome that can efficiently degrade cell walls [94]. Results suggest that midgut microbiota might play key roles in food ingestion and insecticide/xenobiotic metabolism.

## Conclusions

The transcriptome and microbiota data from the *P*. *americana* midgut was obtained via several techniques. Genes related to digestion (eleven genes), detoxification (thirty-seven genes) and oxidative stress response (sixteen genes) were identified. Induction expression analysis revealed that four genes (CYP6K1, alpha-amylase, beta-glucosidase and aminopeptidase) were upregulated more than 10.0-fold in response to insecticide pressure. Tissue expression profiles implied that the selected detoxification enzymes were midgut- and fat body-biased. In addition, the expression of digestive enzymes was found to be much higher in the midgut than in other tissues. The midgut microbiota was found to contain primarily four phyla: Firmicutes, Bacteroidetes, Actinobacteria and Proteobacteria. The microbiota organisms that correlated with digestion, detoxification or oxidative stress response were found to include orders Clostridiales, Lactobacillales and Burkholderiales. These results may provide important information for us to understand the high capacity to adapt to complex environments in *P*. *americana*.

## Supporting Information

S1 Fig Amino acid sequence alignment of three putative P450s with other species. Identical amino acids are shaded in grey for 80% similarity and black for 100% similarity. The ‘■’ indicated the heme-binding site, ‘★’ indicated the meander region, magenta region (conserved sequences ‘ETLR’) showed the conservative sequence of CYP6 family, ‘▼’ indicated the characteristic structural unit of helix I, blue region (conserved sequences ‘EVDTFMFEGHDTT’) showed the conservative sequence of CYP4 family and ‘●’ represented the N-terminal conservative sequence of helix C. Zn: *Zootermopsis nevadensis*; Pa: *Periplaneta americana*. ZnCYP4C1 (Acc. Number: KDR11277.1); ZnCYP6K1 (Acc. Number: KDR14071.1); ZnCYP6J1 (Acc. Number: KDR14072.1).
(TIF)

S2 Fig Amino acid sequence alignment of putative GSTD with other species. Identical amino acids are shaded in grey for 80% similarity and black for 100% similarity. The ‘★’ indicated the catalytic residue Ser. Residues involved in binding glutathione (G-site) were marked with G and those forming the hydrophobic site (H-site) with H. The secondary-structure elements were underlined and labelled (α-helices starting with α and β-strands with β). Bg: *Blattella germanica*; Cp: *Cryptocercus punctulatus*; Lm: *Locusta migratoria*; Pa: *Periplaneta americana*. BgGSTD (Acc. Number: AEV23880.1); CpGSTD1 (Acc. Number: AFK49803.1); LmGSTD (Acc. Number: ADR30117.1).
(TIF)

S3 Fig Amino acid sequence alignment of putative alpha-amylase with other species. Identical amino acids are shaded in grey for 80% similarity and black for 100% similarity. The ‘■’ indicated the active site, ‘★’ indicated the catalytic site and ‘▲’ indicated the Ca-binding site. Bg: *Blattella germanica*; Rs: *Reticulitermes speratus*; Zn: *Zootermopsis nevadensis*; Pa: *Periplaneta americana*. Bgα-amylase (Acc. Number: ABC68516.1); Rsα-amylase (Acc. Number: AGJ52072.1); Znα-amylase (Acc. Number: KDR10404.1).
(TIF)

S4 Fig Amino acid sequence alignment of putative beta-glucosidase with other species. Identical amino acids are shaded in grey for 80% similarity and black for 100% similarity. The ‘★’ indicated the amino acid residue of active site. Blue region showed the conservative amino acid residue. Nt: *Nasutitermes takasagoensis*; Nk: *Neotermes koshunensis*; Ps: *Panesthia angustipennis spadica*; Pa: *Periplaneta americana*. Ntβ-glucosidase (Acc. Number: BAI50023.1); Nkβ-glucosidase (Acc. Number: BAB91145.1); Psβ-glucosidase (Acc. Number: BAU51446.1).
(TIF)

S5 Fig Amino acid sequence alignment of putative aminopeptidase with other species. Identical amino acids are shaded in grey for 80% similarity and black for 100% similarity. The ‘▲’indicated the Zinc-metalloprotease domain (HEXXH), ‘★’ indicated the Zn binding site, ‘■’ indicated the N-glycosylation sites and ‘●’indicated the O-glycosylation sites. Bi: *Bombus impatiens*; Hs: *Harpegnathos saltator*; Zn: *Zootermopsis nevadensis*; Pa: *Periplaneta americana*. Bi aminopeptidase (Acc. Number: XP_003487612.1); Hs aminopeptidase (Acc. Number: EFN87052.1); Zn aminopeptidase (Acc. Number: KDR22502.1).
(TIF)

S1 Table Primers used in qRT-PCR. (DOC)

S2 Table Length distribution of contigs and unigenes in the *P*. *americana* midgut transcriptome. (DOCX)

S3 Table Percentage of homologous hits in the *P*. *americana* midgut transcriptome to other insects. Species which have more than 1.0% matching hits are shown.
(DOCX)
