# Supplementary figures and images for: Midgut Transcriptome of the Cockroach Periplaneta americana and Its Microbiota: Digestion, Detoxification and Oxidative Stress Response
Source: PLoS One. 2016 May 6;11(5):e0155254. doi: 10.1371/journal.pone.0155254 (PMC4859610; doi:10.1371/journal.pone.0155254)

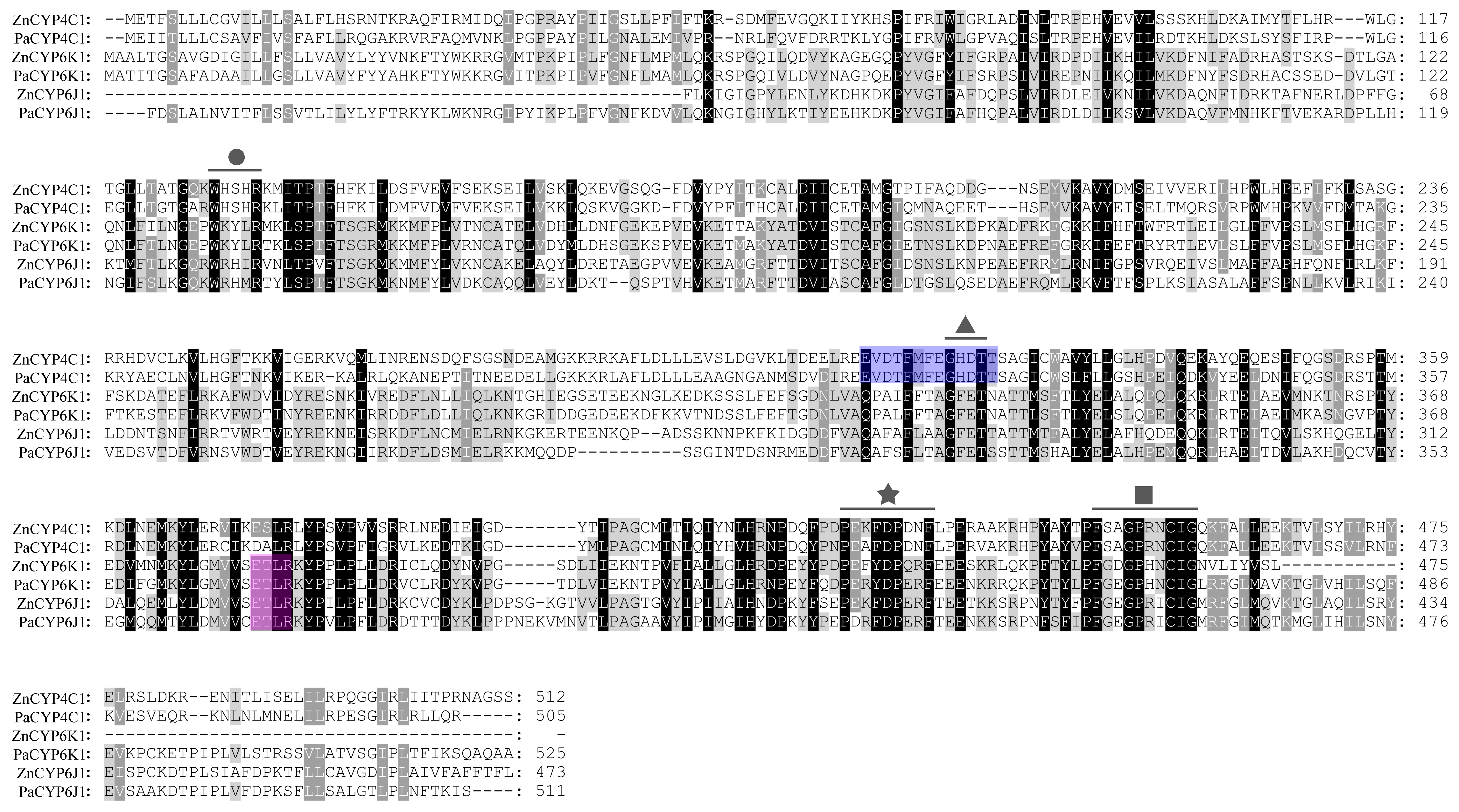

Supplement: S1 Fig — Identical amino acids are shaded in grey for 80% similarity and black for 100% similarity. The ‘■’ indicated the heme-binding site, ‘★’ indicated the meander region, magenta region (conserved sequences ‘ETLR’) showed the conservative sequence of CYP6 family, ‘▼’ indicated the characteristic structural unit of helix I, blue region (conserved sequences ‘EVDTFMFEGHDTT’) showed the conservative sequence of CYP4 family and ‘●’ represented the N-terminal conservative sequence of helix C. Zn: Zootermopsis nevadensis; Pa: Periplaneta americana. ZnCYP4C1 (Acc. Number: KDR11277.1); ZnCYP6K1 (Acc. Number: KDR14071.1); ZnCYP6J1 (Acc. Number: KDR14072.1). (TIF) [file pone.0155254.s001.tif]

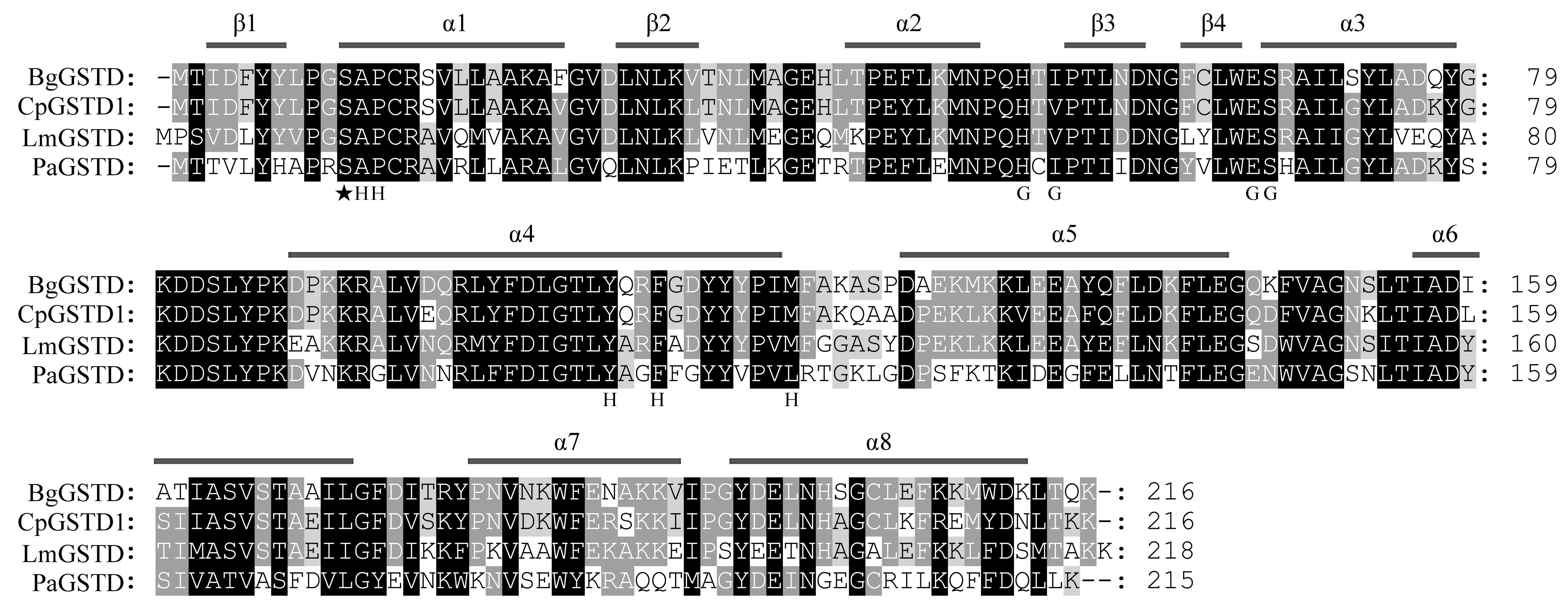

Supplement: S2 Fig — Identical amino acids are shaded in grey for 80% similarity and black for 100% similarity. The ‘★’ indicated the catalytic residue Ser. Residues involved in binding glutathione (G-site) were marked with G and those forming the hydrophobic site (H-site) with H. The secondary-structure elements were underlined and labelled (α-helices starting with α and β-strands with β). Bg: Blattella germanica; Cp: Cryptocercus punctulatus; Lm: Locusta migratoria; Pa: Periplaneta americana. BgGSTD (Acc. Number: AEV23880.1); CpGSTD1 (Acc. Number: AFK49803.1); LmGSTD (Acc. Number: ADR30117.1). (TIF) [file pone.0155254.s002.tif]

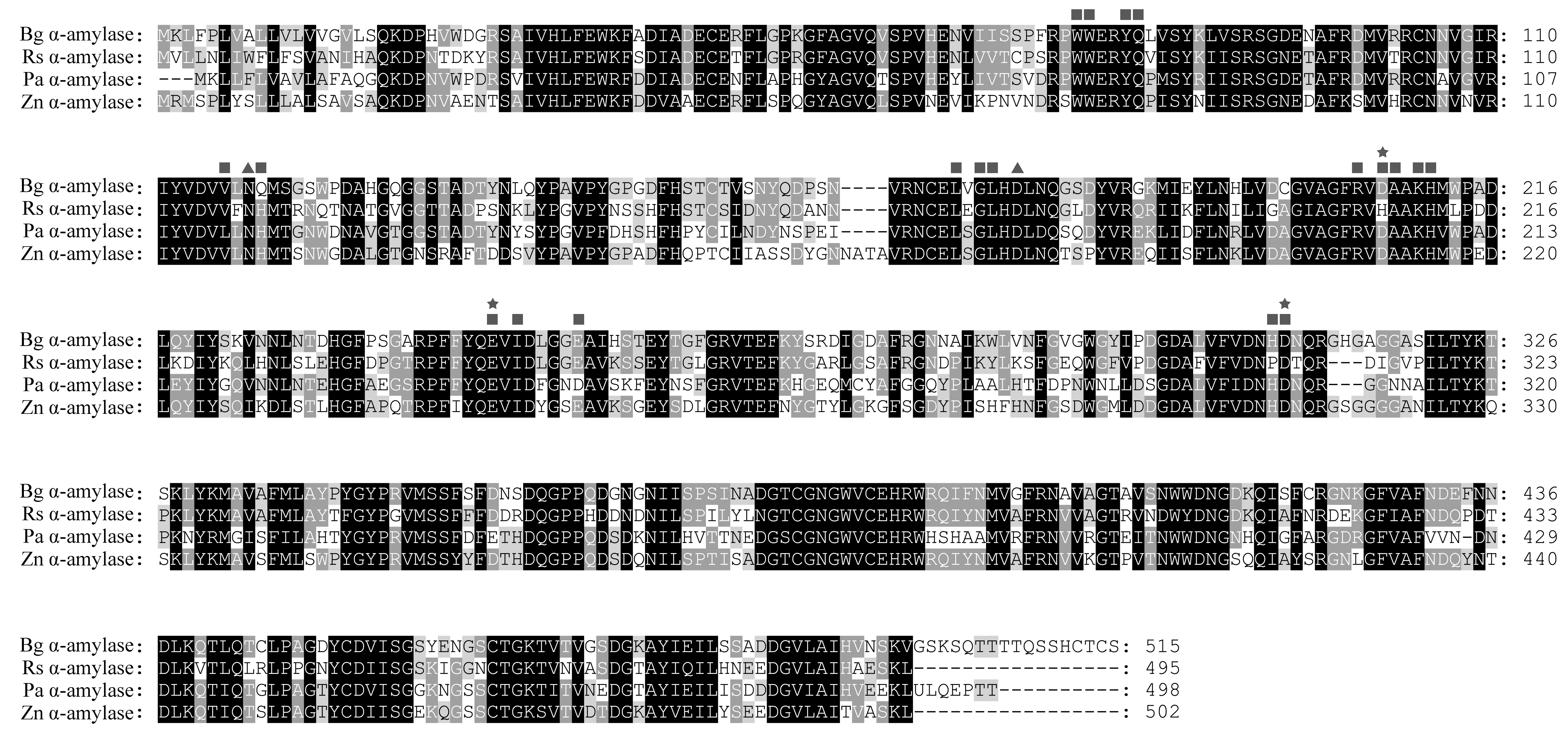

Supplement: S3 Fig — Identical amino acids are shaded in grey for 80% similarity and black for 100% similarity. The ‘■’ indicated the active site, ‘★’ indicated the catalytic site and ‘▲’ indicated the Ca-binding site. Bg: Blattella germanica; Rs: Reticulitermes speratus; Zn: Zootermopsis nevadensis; Pa: Periplaneta americana. Bgα-amylase (Acc. Number: ABC68516.1); Rsα-amylase (Acc. Number: AGJ52072.1); Znα-amylase (Acc. Number: KDR10404.1). (TIF) [file pone.0155254.s003.tif]

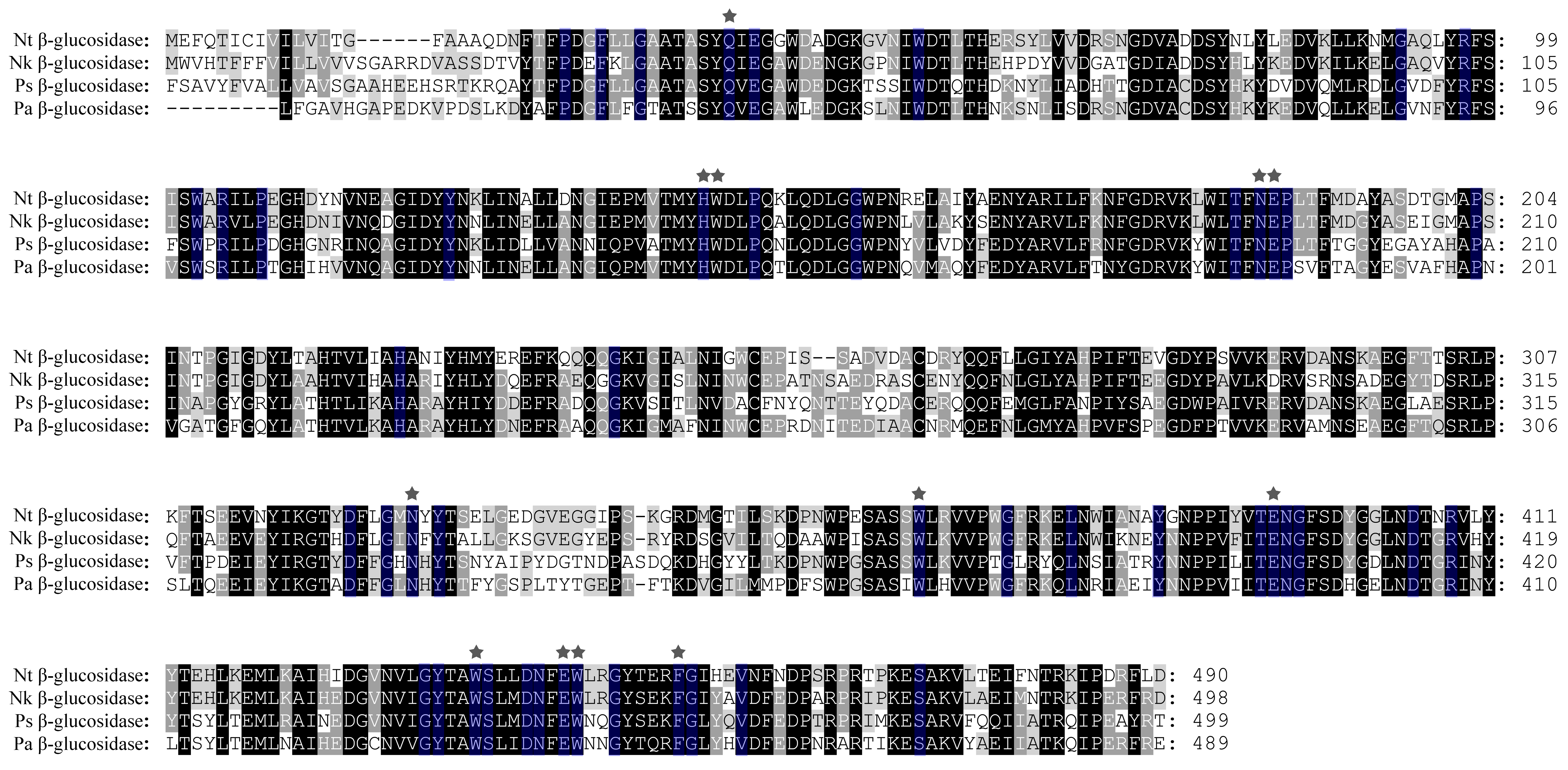

Supplement: S4 Fig — Identical amino acids are shaded in grey for 80% similarity and black for 100% similarity. The ‘★’ indicated the amino acid residue of active site. Blue region showed the conservative amino acid residue. Nt: Nasutitermes takasagoensis; Nk: Neotermes koshunensis; Ps: Panesthia angustipennis spadica; Pa: Periplaneta americana. Ntβ-glucosidase (Acc. Number: BAI50023.1); Nkβ-glucosidase (Acc. Number: BAB91145.1); Psβ-glucosidase (Acc. Number: BAU51446.1). (TIF) [file pone.0155254.s004.tif]

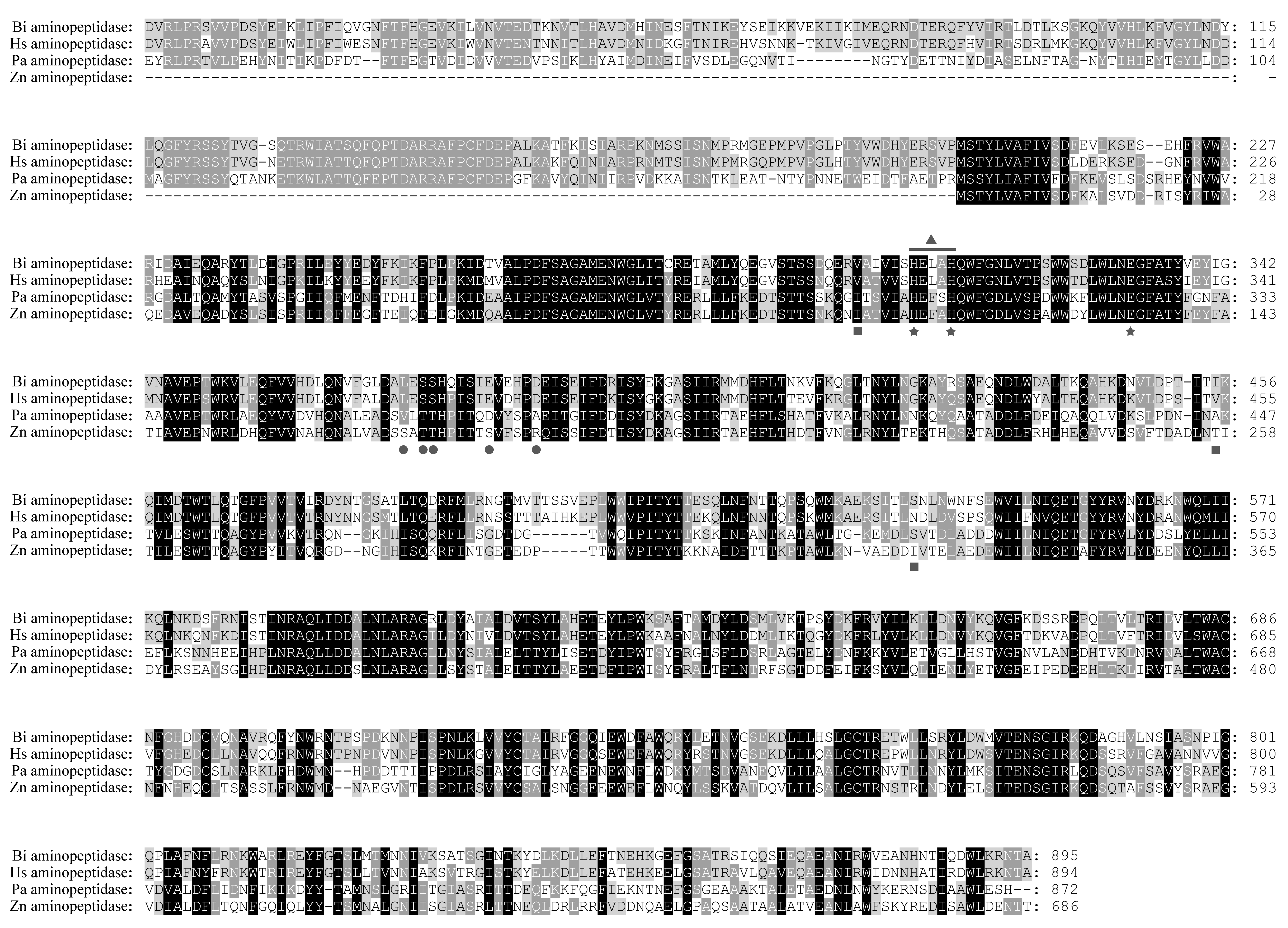

Supplement: S5 Fig — Identical amino acids are shaded in grey for 80% similarity and black for 100% similarity. The ‘▲’indicated the Zinc-metalloprotease domain (HEXXH), ‘★’ indicated the Zn binding site, ‘■’ indicated the N-glycosylation sites and ‘●’indicated the O-glycosylation sites. Bi: Bombus impatiens; Hs: Harpegnathos saltator; Zn: Zootermopsis nevadensis; Pa: Periplaneta americana. Bi aminopeptidase (Acc. Number: XP_003487612.1); Hs aminopeptidase (Acc. Number: EFN87052.1); Zn aminopeptidase (Acc. Number: KDR22502.1). (TIF) [file pone.0155254.s005.tif]
