# Supplementary material for: Midgut Transcriptome of the Cockroach Periplaneta americana and Its Microbiota: Digestion, Detoxification and Oxidative Stress Response
Source: PLoS One. 2016 May 6;11(5):e0155254. doi: 10.1371/journal.pone.0155254 (PMC4859610; doi:10.1371/journal.pone.0155254)
Supplement: S1 Table — (DOC) [file pone.0155254.s006.doc]

**Table S1.** Primers used in qRT-PCR.

| Gene name | Forward primer | Reverse primer |
| --- | --- | --- |
| CYP6J1 | AAGAGACTATGGCACGCTTCACC | ACACCTTGAGCAGATTGGGAGAGA |
| CYP4C1 | CCTCGCAATCGTCTGTTCCAAGTG | GCCAGGGTCGGATGAAGGAGTAAG |
| CYP6K1 | ACTGGTGGCGGTGTACTTCTACT | CCAACATAAGGCTCTTGCGGTCC |
| GST Delta | CGCAGGCTTCTTCGGCTATTACG | CGACGGTGGCAACAATGGAGTAG |
| Alpha-amylase | CGACCACAGCCACTTCCACC | CATCCACCCTAAAGCCAGCAACTC |
| Beta-glucosidase | TCACGCACAACAAGTCCAACCT | TCAATTCCAGCCTGATTCACAACG |
| Aminopeptidase | CTTGGCGTCTTGCTGAACAGTATG | AACGAAGGTCGCATGTGACAGAA |
| β-actin | TTACCACCACTGCCGAACGA | CCTCTGGACAACGGAACCTC |
| GAPDH | ATGCTGTCACTGCAACACAGAAGA | CAGGAACACGGAATGCCATACCA |
