# Supplementary material for: Midgut Transcriptome of the Cockroach Periplaneta americana and Its Microbiota: Digestion, Detoxification and Oxidative Stress Response
Source: PLoS One. 2016 May 6;11(5):e0155254. doi: 10.1371/journal.pone.0155254 (PMC4859610; doi:10.1371/journal.pone.0155254)
Supplement: S2 Table — (DOCX) [file pone.0155254.s007.docx]

**Table S2.** Length distribution of contigs and unigenes in the *P. americana* midgut transcriptome.

| Sequence size (nt) | Number of contigs | Number of unigenes |
| --- | --- | --- |
| 100-500 | 146624 | 63658 |
| 500-1000 | 9833 | 11614 |
| 1000-1500 | 2756 | 3698 |
| 1500-2000 | 1252 | 1741 |
| ＞2000 | 1356 | 2194 |
