# Supplementary material for: Midgut Transcriptome of the Cockroach Periplaneta americana and Its Microbiota: Digestion, Detoxification and Oxidative Stress Response
Source: PLoS One. 2016 May 6;11(5):e0155254. doi: 10.1371/journal.pone.0155254 (PMC4859610; doi:10.1371/journal.pone.0155254)
Supplement: S3 Table — Species which have more than 1.0% matching hits are shown. (DOCX) [file pone.0155254.s008.docx]

**Table S3.** Percentage of homologous hits in the *P. americana* midgut transcriptome to other insects. Species which have more than 1.0% matching hits are shown.

| Species | Percentage of homologous hits |
| --- | --- |
| *Tribolium castaneum* | 11.96% |
| *Pediculus humanus corporis* | 10.11% |
| *Megachile rotundata* | 5.83% |
| *Acyrhosiphum pisum* | 5.24% |
| *Nasonia vitripennis* | 4.99% |
| *Camponotus floridana* | 4.44% |
| *Harpegnathos saltator* | 3.98% |
| *Acromyrmex echinatior* | 3.00% |
| *Bombus impatiens* | 2.72% |
| *Apis florea* | 2.24% |
| *Apis mellifera* | 2.24% |
| *American monarch* | 2.17% |
| *Bombus terrestri* | 2.05% |
| *Daphnia pulex* | 1.88% |
| *Solenopsis invicta* | 1.68% |
| *Aedes aegypti* | 1.60% |
| *Bombyx mori* | 1.56% |
| *Strongylocentrotus purpuratus* | 1.29% |
| *Culex pipiens quinquefasciatus* | 1.28% |
| *Galendromus occidentalis* | 1.25% |
| *Hydra magnipapillata* | 1.18% |
| *Anopheles gambiae* | 1.06% |
| others | 26.25% |
